# Persistence of *Mycobacterium tuberculosis* in response to infection burden and host-induced stressors

**DOI:** 10.3389/fcimb.2022.981827

**Published:** 2022-12-02

**Authors:** Trisha Parbhoo, Haiko Schurz, Jacoba M. Mouton, Samantha L. Sampson

**Affiliations:** Department of Science and Technology (DSI)- National Research Foundation (NRF) Centre of Excellence for Biomedical Tuberculosis Research (CBTBR), South African Medical Research Council Centre (SAMRC) Centre for Tuberculosis Research, Division of Molecular Biology and Human Genetics, Faculty of Medicine and Health Sciences, Stellenbosch University, Cape Town, South Africa

**Keywords:** *Mycobacterium tuberculosis*, persistence, persisters, bacterial heterogeneity, host-pathogen interaction, phagocytosis, phagosome acidification

## Abstract

**Introduction:**

As infection with Mycobacterium tuberculosis progresses, the bacilli experience various degrees of host stressors in the macrophage phagosome such as low pH, nutrient deprivation, or exposure to toxic agents, which promotes cell-to-cell phenotypic variation. This includes a physiologically viable but non- or slowly replicating persister subpopulation, which is characterised by a loss of growth on solid media, while remaining metabolically active. Persisters additionally evade the host immune response and macrophage antimicrobial processes by adapting their metabolic pathways to maintain survival and persistence in the host.

**Methods:**

A flow cytometry-based dual-fluorescent replication reporter assay, termed fluorescence dilution, provided a culture-independent method to characterize the single-cell replication dynamics of M. tuberculosis persisters following macrophage infection. Fluorescence dilution in combination with reference counting beads and a metabolic esterase reactive probe, calcein violet AM, provided an effective approach to enumerate and characterize the phenotypic heterogeneity within M. tuberculosis following macrophage infection.

**Results:**

Persister formation appeared dependent on the initial infection burden and intracellular bacterial burden. However, inhibition of phagocytosis by cytochalasin D treatment resulted in a significantly higher median percentage of persisters compared to inhibition of phagosome acidification by bafilomycin A1 treatment.

**Discussion:**

Our results suggest that different host factors differentially impact the intracellular bacterial burden, adaptive mechanisms and entry into persistence in macrophages.

## Introduction

Macrophages represent the first line of defence against *Mycobacterium tuberculosis*, and recognize invading bacteria *via* cell surface receptors [e.g. pattern recognition receptors (PRRs), complement receptors and antibody binding *via* Fc receptors]. This process initiates a series of dynamic host antimicrobial strategies, such as phagocytosis and phagosome acidification, for bacterial engulfment, and antigen presentation (Sia and Rengarajan, 2019).

The phagocytic process is initiated upon pathogen recognition of *M. tuberculosis* pathogen-associated molecular patterns (PAMPs) by host PRRs. *M. tuberculosis* PAMPs include the cell wall components lipoarabinomannan (LAM), mannose-capped lipoarabinomannan (ManLAM), phthiocerol dimycocerosate (PDIM) and mycolic acids, which possess an array of ligands and antigenic moieties that facilitate host recognition in addition to providing a complex lipid structure that enables protection from host defence mechanisms (Stamm et al., 2015). Maturation of the phagosome into the acidic phagolysosome represents an essential host process for degradation of invading microbes by enhancing the antimicrobial capacity and immune signalling processes of macrophages (Rohde et al., 2012). This exposes *M. tuberculosis* to the oxidative and lytic activities of reactive oxygen species (ROS), nitric oxide (NO), and low pH through activity of proton-pumping vacuolar-ATPase (V-ATPase) complexes.

*M. tuberculosis* has the remarkable ability to persist in the host for long periods of time by undergoing growth arrest and metabolic adaptation in preparation for long-term persistence (Zimmermann et al., 2017; Maurya et al., 2018). Persisters are defined here in accordance with the definition by Balaban et al. (2019), whereby persisters represent a subpopulation of non- or slowly replicating drug-tolerant bacteria within an isogenic population (Balaban et al., 2019). Clinically, this population has been regarded as a pre-existing drug-tolerant population that is actively enriched for during cell division, or during adaption to host stressors prior to antibiotic treatment (Garton et al., 2008; Jain et al., 2016). Persisters remain undetected by current diagnostic tests, yet provide a reservoir for infection relapse during favourable growth conditions (Kana et al., 2008; Chengalroyen et al., 2016).

In this study, calcein violet acetoxymethyl (CV-AM) was utilized as a marker for metabolic esterase activity. CV-AM has previously been utilized for mammalian systems (Lis et al., 2011), and more recently applied to *M. tuberculosis* (Hendon-Dunn et al., 2016; Mishra et al., 2019). Esterase activity, as measured by CV-AM, is suggested to facilitate survival by providing an important intracellular source of energy and carbon for *M. tuberculosis* (**Supplementary Table 1**). Many *M. tuberculosis* esterases/lipases appear to be upregulated during stress, suggesting the importance of these lipolytic enzymes during adaptation to intracellular survival and persistence (Ghazaei, 2018). CV-AM will thus provide a measure of intracellular enzyme hydrolysis and substrate reactivity of esters containing short-medium chain fatty acids.

A flow cytometry-based technique, termed fluorescence dilution, provided a culture-independent method to characterize the single-cell replication dynamics of *M. tuberculosis* persisters following macrophage infection (Mouton et al., 2016). Fluorescence dilution was utilized in combination with flow cytometry and CV-AM staining to provide insight into the heterogeneous nature of persisters, and for characterization of esterase activity in differentially replicating subpopulations. We further aimed to investigate whether varying infection burdens, and inhibition of phagocytosis and phagosome acidification influences the abundance of persisters internalized by macrophages.

## Material and methods

### Bacterial strains and plasmids

All reagents were purchased from Sigma-Aldrich (St. Louis, MO, USA), unless otherwise specified. All strains and plasmids utilized in this study is listed in **Table 1**.

**Table 1:** Strains and plasmids utilized in this study.

	Relevant characteristics	Source/Reference
**Bacterial strains**		
*M. tuberculosis* H37Rv	Pathogenic lab strain	ATCC 27294
*M. tuberculosis* Δ*leuD*Δ*panCD*	Double leucine and pantothenate auxotroph, Hyg^R^	Sampson et al., 2004
**Plasmids**		
pTiGc	Dual fluorescent reporter construct, Kan^R^ Constitutive GFP, TurboFP635 under control of the theophylline inducible riboswitch	Mouton et al., 2016
pST5552	Bacterial Expression vector, Kan^R^ GFP under control of the theophylline inducible riboswitch	Addgene, USA (Plasmid #36255); Seeliger et al., 2012
pSTCHARGE	Bacterial Expression vector, Kan^R^ TurboFP635 under control of the theophylline inducible riboswitch	Mouton et al., 2016

Kan^R^, Kanamycin resistance; Hyg^R^, Hygromycin resistance.

*Mycobacterium tuberculosis* H37Rv (ATCC, 27294) was cultured in Middlebrook 7H9 media supplemented with 10% OADC (oleic acid-albumin-dextrose-catalase supplement), 0.2% (v/v) glycerol and 0.05% (w/v) Tween-80 (7H9-OGT). An attenuated strain of *M. tuberculosis*Δ*leuD*Δ*panCD*, as previously constructed (Sampson et al., 2004), was grown at 37°C in 7H9-OGT supplemented with 50 μg/ml leucine and 24 μg/ml pantothenate, with shaking at 180 rpm, until an optical density at 600 nm (OD_600nm_) of 0.8 (≈ 8x10^7^ CFU/ml) was reached. Selective antibiotics, kanamycin and hygromycin (Thermo Scientific, USA) was added to the cultures when required, at a final concentration of 25 µg/ml and 50 µg/ml, respectively.

For induction of fluorescent proteins under control of the riboswitch promoter, 7H9-OGT was supplemented with 4 mM theophylline for 7 days during culturing.

### Mammalian cell culture

RAW 264.7 (ATCC TIB-71) murine macrophages were cultured in Dulbecco’s Modified Eagle’s Medium (DMEM), supplemented with 10% heat-inactivated fetal bovine serum (FBS) and incubated at 37°C in 5% CO_2_ until 80% confluent. Cells were passaged every 2-3 days or upon reaching 80% confluency, at a ratio of 1:6 in DMEM-10% FBS (D10) and incubated at 37°C in 5% CO_2_.

Twenty hours prior to infection, 48-well plates were seeded with 2.5x10^5^ macrophages per well, and incubated overnight (37°C, 5% CO_2_). Non-adherent macrophages were removed the following day by gentle washing with D10, thereafter macrophages were immunologically activated by supplementing D10 with 100 ng/ml lipopolysaccharide (LPS), and incubated at 37°C in 5% CO_2_ for 60 min.

Single-cell suspensions of early-exponentially replicating *M. tuberculosis* and *M. tuberculosis* Δ*leuD*Δ*panCD* were prepared for infection by brief sonication in an ultrasonic waterbath (UC-1D, Zeus Automation, South Africa) at 37 kHz for 12 min, followed by filtering through a 40 μm cell strainer (Corning, USA) to reduce cell clumping. *M. tuberculosis* cultures were washed twice in D10 supplemented with 2 mM theophylline and added to the macrophage monolayers at a multiplicity of infection (MOI) of 1:1, 5:1 or 10:1 to obtain varying infection burdens, and incubated for 3 hours (37°C, 5% CO_2_). Following bacterial uptake, monolayers were washed with PBS, and the media replaced with D10 containing 2 mM theophylline and 100 U/ml penicillin-streptomycin. Cells were incubated for 60 min (37°C, 5% CO_2_) for removal of any extracellular, non-phagocytosed bacteria. Cells were washed three times with PBS, followed by the addition of fresh D10 containing 2 mM theophylline. Theophylline was retained in the media for 24 hours to maintain expression of TurboFP635 under control of the riboswitch-based promoter; this ensured that dilution of fluorescence was detectable over 5 days. The day 0 time-point refers to harvesting of intracellular bacteria following treatment with 100 U/ml penicillin-streptomycin treatment on the day of infection. Treatment with penicillin-streptomycin was performed daily for 5 days. For *in vitro* measurements, bacterial suspensions was added to wells at the time of infection.

Recovery of intracellular bacteria for flow cytometry involved lysing the macrophages with 500 µl distilled sterile water, whereby plates were incubated for 5 min at room temperature to release the intracellular bacteria. For recovery of whole infected macrophages, macrophages were loosened with 100 μl Accutase, and incubated for 10 min at room temperature for optimal detachment. Infected macrophages or intracellular bacteria were transferred to 2 ml screw cap tubes, washed with Hanks’ Balanced Salt Solution (HBSS), and transferred to 5 ml polypropylene flow cytometer tubes *via* 35 μm cell strainer caps (Corning, USA) prior to flow cytometric analysis. Where required, cells were fluorescently stained (described below), followed by fixation in 4% formaldehyde (v/v) for 30 min.

### Inhibitors of phagocytosis and phagosome acidification

Cytochalasin D (CytD) and bafilomycin A1 (BafA1) were used to inhibit phagocytosis and phagosome acidification, respectively. Inhibitors were hydrated in dimethylsulfoxide (DMSO) for preparation of 1 mM CytD and 20 μM BafA1 master stocks, and stored at -20°C. The minimum optimal concentration for CytD and BafA1 were experimentally determined. To confirm that the DMSO concentration in CytD (0.607% DMSO) and BafA1 (0.05% DMSO) was not detrimental to macrophages, inhibitors were added to uninfected macrophages as a control. Macrophage viability was unaffected throughout infection, as assessed using an MTT assay (data not shown).

Macrophages were pre-treated with 100 ng/ml LPS for 60 min, and following 20 min of treatment, 6 μM CytD or 10 nM BafA1 was added to monolayers and incubated at 37°C in 5% CO_2_ for 40 min. Monolayers were washed with PBS, and the media replaced with D10 containing 2 mM theophylline and either CytD or BafA1. *M. tuberculosis*Δ*leuD*Δ*panCD*::pTiGc, pre-induced with 4 mM theophylline 7 days prior to infection, was added to monolayers at MOI 10:1, and incubated for 3 hours (37°C, 5% CO_2_). The infection procedure was carried out as described before. Importantly, the possibility of bacterial reuptake following CytD treatment would have been minimized by daily removal of extracellular bacteria by penicillin-streptomycin treatment. Following the penicillin-streptomycin treatment, BafA1 was re-added to the fresh D10 media each day for 5 days. Uninfected macrophages treated with each inhibitor served as a negative control, and was maintained throughout the 5-day infection. Intracellular bacteria were harvested, fluorescently stained, formaldeyhyde-fixed and prepared for flow cytometric analysis.

### Calcein violet-acetoxymethyl

CV-AM (Invitrogen, Life Technologies, USA) is a lipophilic non-fluorescent dye able to permeate the cell membranes of live cells, whereby intracellular esterases cleave the AM group to allow fluorescence. The fluorescent intensity is proportional to the level of functional esterases.

CV-AM was freshly prepared for each experiment by reconstituting a pre-warmed vial in DMSO to prepare a 1 μg/μl working stock. *M. tuberculosis* from *in vitro* cultures or recovered from lysed macrophages was stained with 5 ng/µl CV-AM in 500 μl HBSS for 30 min at 37°C with shaking. Samples were fixed in 4% formaldehyde for 30 min, resuspended in HBSS, and transferred to 5 ml polypropylene flow cytometer tubes *via* a 35 μm cell strainer cap (Corning, USA) for flow cytometric acquisition.

*M. tuberculosis*::pTiGc was cultured as described above, till OD_600nm_ = 0.5. Cultures were then inoculated at an OD_600nm_ = 0.05, following which the OD_600nm_ was assessed every 2 days for 21 days. Following sampling, an aliquot of culture was resuspended in HBSS to OD_600nm_ = 0.5 (≈ 5x10^7^ bacteria/ml) and stained with CV-AM to ensure a constant dye:cell distribution. To acquire dead cells for the control sample, 1 ml of cells was heat-killed at 95°C for 60 min, stained with CV-AM and formaldeyhyde-fixed prior to flow cytometric acquisition.

### pHrodo Green STP ester

To confirm inhibition of phagosome acidification following BafA1 treatment, the pH responsive fluorescent probe, pHrodo Green STP ester (Life Technologies, USA) was utilised. pHrodo Green was hydrated in DMSO to generate 1 mM master stocks, and stored at -20°C. Pre-induced *M. tuberculosis*Δ*leuD*Δ*panCD*::pSTCHARGE with 4 mM theophylline for 7 days was adjusted to OD_600nm_ = 1, washed twice with HBSS before resuspending in a 10^th^ of the total volume of 100 mM sodium bicarbonate to stabilize the pH. Cells were stained with 0.5 mM pHrodo Green for 60 min at room temperature, with shaking. Following staining, cells were resuspended in HBSS to 1 ml and washed three times in 1 ml HBSS. To maintain fluorescence of TurboFP635, pHrodo-labelled cells were resuspended in 1 ml 7H9-OGT media containing 4 mM theophylline prior to macrophage infection.

To determine the pH response range of the conjugate, pre-induced pHrodo Green-labelled *M. tuberculosis*Δ*leuD*Δ*panCD*::pSTCHARGE with 4 mM theophylline for 7 days was diluted 1:5 to reach OD_600nm_ = 0.2 and exposed to 50 mM potassium phosphate buffers ranging from pH 4.5-7.5. Neutral pH conditions were reflected by non-fluorescence, and increasing fluorescent intensity was observed with increasing acidity. Samples were serially diluted in a black, clear bottom 96-well plate (Corning, USA), and fluorescence readings were taken to determine the signal limit of detection using the FLUOstar Omega 96-well microplate reader (BMG Labtech, Germany). Optical settings were applied using a 584/640 nm and 485/520 nm filter for excitation of TurboFP635 and pHrodo Green, respectively. To limit background noise, the auto-gain adjustment parameter was applied to the positive single-colour controls.

Fifty mM potassium phosphate buffer (pH 4.5-7.5) and 100 mM sodium bicarbonate (pH 8.5) were prepared in distilled water and the pH confirmed using a calibrated pH probe prior to usage. Buffers were filter-sterilized and stored at room temperature until use.

### Flow cytometric acquisition

The BD fluorescence-activated cell sorter (FACS) Jazz (Becton Dickinson Biosciences, USA) was used for *M. tuberculosis* sample analysis. To calibrate fluorescence in each detector of the laser, quality control was performed using the 8-peak quality control beads (BD Biosciences, USA).

**Figure 1:**
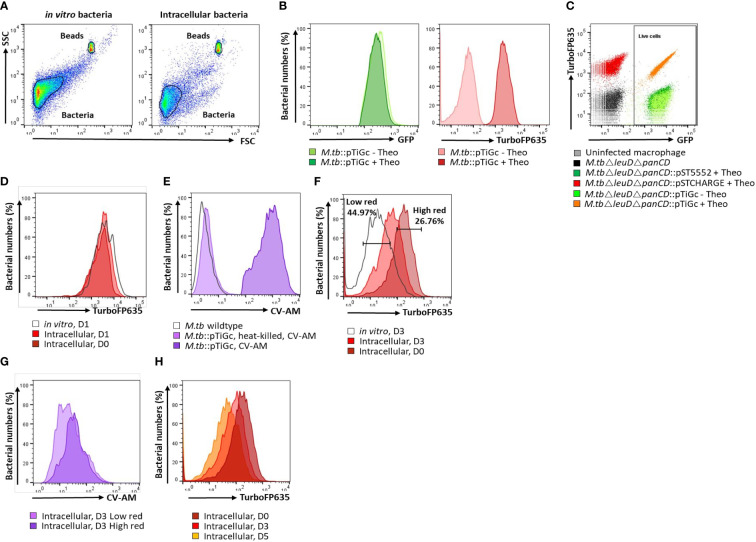
Flow cytometry gating strategy. **(A)** A primary gate was applied to the bacterial population according to forward scatter (FSC) and side scatter (SSC) properties. For cell enumeration, a secondary gate was applied to the non-fluorescent bead population. **(B)** *M. tuberculosis*Δ*leuD*Δ*panCD*::pTiGc was cultured in the presence of 4 mM theophylline to allow induction of the riboswitch-based promoter for expression of TurboFP635. Constitutive expression of GFP is observed, whilst fluorescence of TurboFP635 is observed following induction with theophylline. **(C)** Selecting on the bacterial population, a rectangle gate was created to select for live cells, according to their GFP positivity. Single colour controls ensured optimal voltage settings for positive fluorescence of GFP (pST5552) and TurboFP635 (pSTCHARGE), above that of the autofluorescence of unstained cells. **(D)** The fluorescence dilution technique allows monitoring of bacterial replication for 5 generations. To improve detection of the fluorescent signal to allow measurement over 5 days, theophylline, was retained in culture for 24 hours. Bacterial replication could thus effectively be monitored from day 2 onwards since the fluorescent signal remained stable *in vitro* and intracellularly between day 0 and day 1. **(E)** Following harvesting of intracellular bacteria, cells were stained with CV-AM for analyses of metabolic esterase activity. CV-AM is a non-polar, cell permeable fluorogenic probe that is rapidly hydrolysed to a polar, fluorescent compound by intracellular esterases of live cells. Dead cells no longer possess esterase activity, and will thus not convert to the fluorescent calcein, whilst calcein is stably retained in live cells. **(F)** Selecting on the live cells population, dilution of the TurboFP635 fluorescent signal provided an indication of bacterial replication following removal of the inducer, theophylline (Theo). The high red gate was created based on maximum TurboFP635 fluorescence observed at D0 using the range tool and used to detect mycobacteria that retain their TurboFP635 fluorescence from later time points (D3 and D5), representing slow or non-growing bacteria. The low TurboFP635 gate was created to distinguish replicating intracellular bacteria, as visually assessed when overlayed with *in vitro* day 3 or day 5 bacteria. **(G)** Selecting on the high and low TurboFP635 subpopulations, the esterase activity of intracellular bacteria was assessed by overlaying on a histogram plot. **(H)** Fluorescence dilution of the TurboFP635 signal over time. The geometric median fluorescent intensity (MFI) of TurboFP635 enabled determination of the number of bacterial generations during infection. CV-AM, calcein violet AM.

A primary gate was set on total bacteria based on forward scatter (FSC) and side scatter (SSC) properties and a second gate selected the reference bead population (See below; Invitrogen, USA) for flow cytometric quantification of samples (**Figure 1**). Fluorescence was acquired using logarithmic scaling in acquisition mode with the detection threshold set on SSC, using the parameters listed in **Table 2**. For each experiment, compensation was performed using unlabelled and single colour controls.

**Table 2:** Excitation and emission properties of fluorescent probes utilized.

Fluorophore (s)	Ex/Em (nm)	Laser (nm)	Bandpass filter (nm)
GFP	408/509	488	530/40
TurboFP635	588/635	561	610/20
CV-AM	400/452	405	450/50
pHrodo Green	505/525	488	530/40

Ex/Em: excitation and emission wavelengths.

Thirty-thousand events were captured for all samples, and included a stopping gate selected on live cells (GFP positive). BD FACS Sortware software version 1.1.0.84 generated raw data, which was subsequently processed and analysed using FlowJo software version 10.0.8 (Tree Star Inc., USA). Dot plots were largely utilized for viability analysis, and for setting up gates to determine positivity in fluorescence. Histogram overlays of plots allowed visual detection of the shift in fluorescent signal, and statistical assessment using the median fluorescent intensity (MFI) of each gated population. The MFI of TurboFP635 at a given time was used to calculate the number of bacterial generations, as previously described (Mouton et al., 2016).

A calibrated suspension of 6 μm non-fluorescent microsphere reference beads (suspended at 1x10^8^ beads/ml) supplied with the BacLight Live/Dead Bacterial Viability and Counting kit (Invitrogen, USA) was sonicated for 10 min and 5 µl (i.e. 5x10^5^ beads) was added to selected samples. The number of reference beads recorded by flow cytometry was used to enumerate bacterial populations using the following formula (Adapted from ThermoFisher Scientific, 2004):


$$\text{Bacteria}/\text{ml}=\frac{\text{Bacterial events}}{\text{Bead events}}\times \frac{\#\text{ Beads per sample}}{\text{Sample volume}}\;\times \text{Bacterial dilution factor}\;$$

### Statistical analysis

Bacterial numbers calculated using the reference beads was quality controlled (QC) and analysed using the R programming environment, version 4.0.3 (RStudio Team, 2020) and GraphPad Prism, version 8.0 (GraphPad Software, USA). QC of the macrophage infection data included removal of samples with outlying *in vitro* and intracellular bacterial counts at day 0, based on visual inspection of box and whisker plots. The cut-off for outliers was determined based on the distribution of the data, whereby data points outside 1.5 times of the interquartile range above the upper and below the lower quartile were removed.

To compare distributions, a one-way repeated measures ANOVA was implemented to detect significant differences in bacterial numbers or MFI (*in vitro* and intracellular) between three or more groups at different time points. Following a significant ANOVA result, a *post hoc* multiple comparison test was done to compare the distribution of data between two groups using the 2-sided (unpaired) Students t-test or Wilcoxon Rank-Sum test with Bonferroni multiple test correction for parametric or non-parametric data, respectively. Distribution of the data was inspected visually for normality using QQ-plots. The significance threshold for all analysis was set to a Bonferroni corrected p-value < 0.05.

To determine the extent of correlation between the infection burden and intracellular bacterial burden, against persister numbers at day 3 and day 5, the Pearson’s product-moment correlation or Kendall Tau correlation test was utilised for parametric or non-parametric data, respectively. The strength of the correlation was assessed using the square of the Pearson product moment correlation coefficient (r^2^) or Kendall’s Tau correlation coefficient (τ) and associated p-value. For a visual comparison, non-linear local regression lines with 95% confidence interval were added to the correlation plots using the R loess function from the nlshelper package (Duursma, 2017).

## Results

### Influence of initial infection burden and subsequent intracellular bacterial burden on persister numbers

The double auxotrophic *M. tuberculosis*Δ*leuD*Δ*panCD* strain utilized in this study provides a model organism for *M. tuberculosis* research, which recapitulates salient features of the physiology, replication dynamics and response to treatment of *M. tuberculosis* H37Rv (Mouton et al., 2019). The attenuated strain is additionally safe for use in biosafety level 2 facilities (Sampson et al., 2004). In previous work, the non- or slowly replicating *M. tuberculosis* persister subpopulation was observed 3 days following macrophage uptake, as identified using fluorescence dilution in combination with flow cytometry (Mouton et al., 2016). The fluorescence dilution reporter, pTiGc, contains a constitutive reporter (GFP) for tracking of viable bacteria, whilst an inducible reporter (TurboFP635) allows measurement of bacterial replication (see gating strategy, **Figure 1**). This approach exploits the principle that following induction, the inducible fluorescent signal will halve with each successive cell division after removal of the inducer, and allows monitoring of bacterial replication for 5 generations. Bacterial cells not undergoing replication or existing in a slowly replicating state, such as persisters, will retain their TurboFP635 fluorescent signal (Helaine et al., 2010). Following flow cytometric detection, the persister subpopulation was confirmed to be antibiotic tolerant following exposure to the antibiotic D-cycloserine. Proportions of persisters were similar in D-cycloserine -treated *vs* non-treated samples, confirming that the non-replicating cells observed with fluorescence dilution are indeed drug-tolerant persisters (Mouton et al., 2016).

To determine whether the number and percent of persisters observed at day 3 and day 5 is influenced by the initial infection burden, macrophages were infected with varying bacterial numbers. Here, initial infection burden refers to the number of bacteria applied to infect the macrophages, and the initial intracellular burden refers to the number of bacteria taken up by macrophages (day 0). Retention of theophylline in the growth media for 24 hours following infection ensured that the fluorescent signal of TurboFP635 would remain detectable by day 5 should initial early replication and TurboFP635 dilution occur (**Figure 1D**). Enumeration of intracellular *M. tuberculosis*Δ*leuD*Δ*panCD* using reference beads in combination with flow cytometry provided an effective culture-free approach for counting heterogeneous populations.

**Figure 2:**
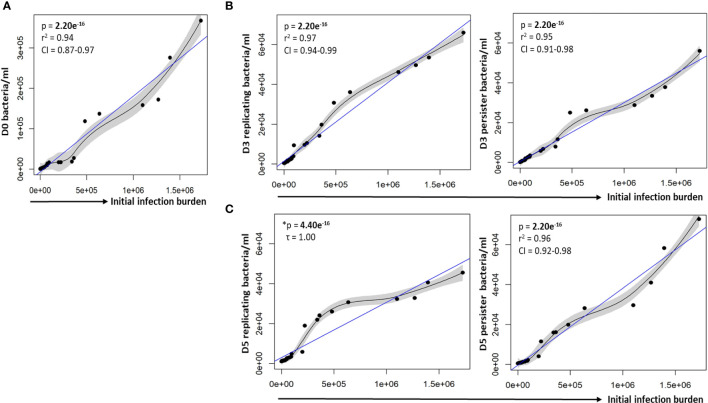
Correlation between the infection burden and growth of intracellular bacteria. **(A)** Macrophages were infected with varying bacterial burdens. Cell enumeration using reference counting beads was used to establish the correlation between the initial infection burden (D0 *in vitro* bacteria/ml) and intracellular bacteria following uptake. Significant positive correlations between the initial infection burden and actively replicating bacteria or persisters was observed at **(B)** day 3 and **(C)** day 5. Data was assessed using the Pearson’s product-moment correlation (linear) and is representative of data independently conducted in 7 biological experiments, including technical triplicates. *The Kendall’s Tau correlation was used to determine the non-linear correlation coefficient tau (τ). Significant p-values (p < 0.05) are shown in bold. The blue line represents the regression line for the correlation analyses (Pearson and Kendall), while the black line and associated shaded area represents the local non-linear regression and 95% confidence interval. r^2^, Pearson’s correlation coefficient squared; CI, 95% confidence interval; SEM, standard error of mean.

**Figure 3:**
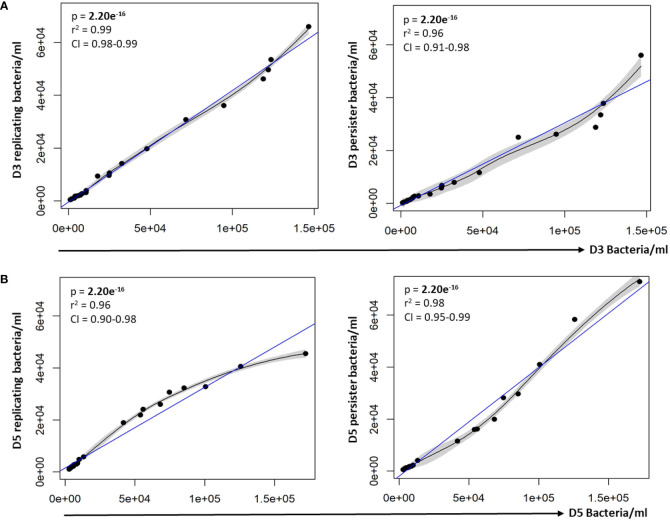
Persister numbers correlated with respective intracellular bacterial numbers. A strong correlation between the infection burden observed at **(A)** day 3 and **(B)** day 5 in relation to the respective persister numbers was observed on the scatter plot. Data was assessed using the Pearson’s product-moment correlation (linear) and is representative of data independently conducted in 7 biological experiments, including technical triplicates. Significant p-values (p < 0.05) are shown in bold. The blue line represents the regression line for the correlation analyses (Pearson), while the black line and associated shaded area represents the local non-linear regression and 95% confidence interval. r^2^, Pearson’s correlation coefficient squared; CI, 95% confidence interval.

To assess whether varying infection burdens influenced intracellular bacterial numbers, the intracellular live bacterial population was enumerated using reference beads, by flow cytometry. The initial infection burden (day 0 *in vitro* bacteria/ml) displayed a significant positive correlation with the intracellular bacterial burden at day 0 (p = 2.20e^-16^; **Figure 2A**). We next assessed the impact of different initial infection and later intracellular bacterial burdens on absolute persister numbers at day 3 and day 5. The initial infection burden significantly correlated with the number of actively replicating bacteria and persisters at day 3 (p-values = 2.20e^-16^; **Figure 2B**) and day 5 (p-values < 2.20e^-16^; **Figure 2C**). Similarly, the intracellular burden significantly correlated with the respective number of actively replicating bacteria and persisters at day 3 (p-values = 2.20e^-16^; **Figure 3A**) and day 5 (p-values = 2.20e^-16^; **Figure 3B**).

**Figure 4:**
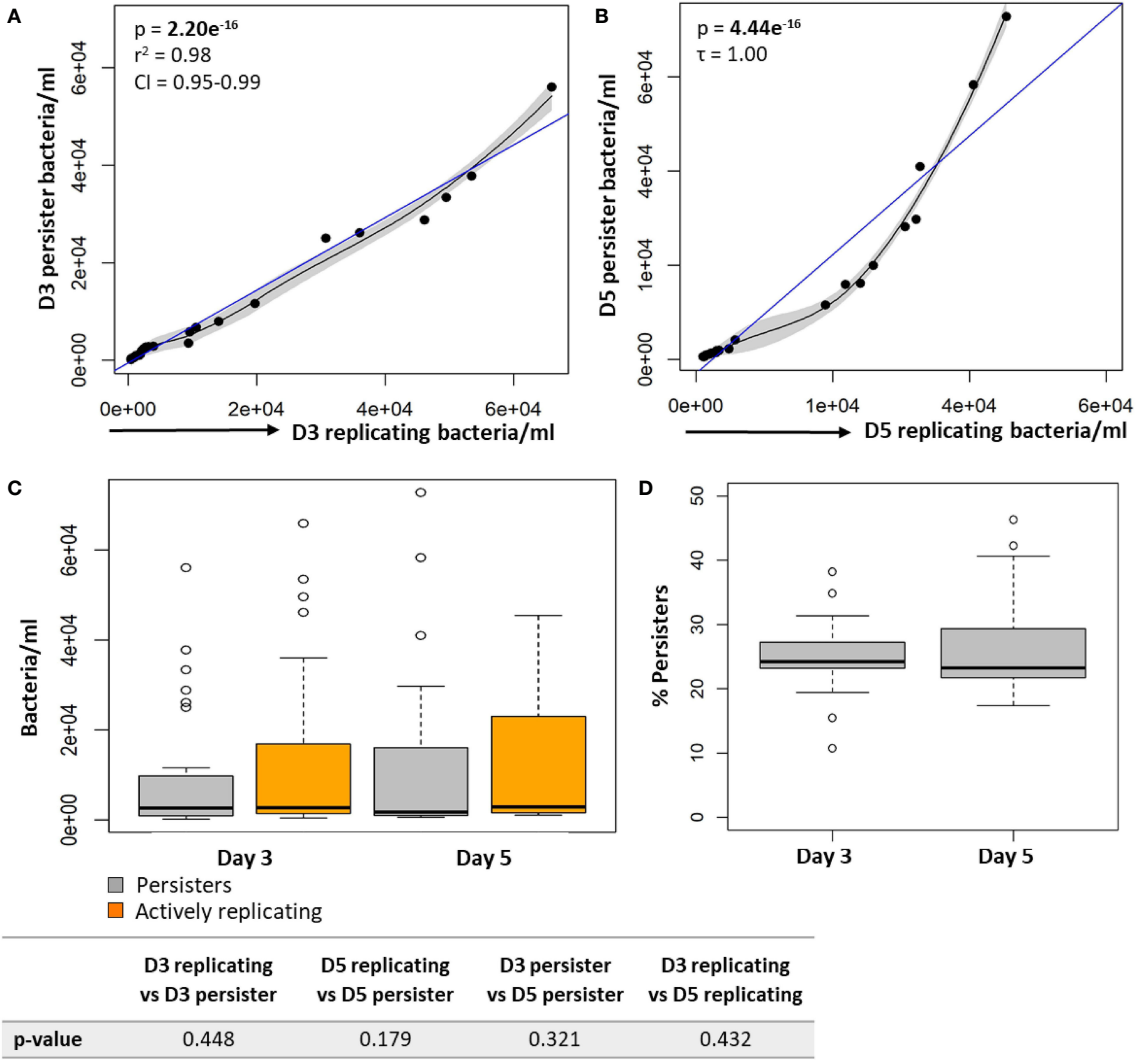
Persister numbers correlated with the number of intracellular growing mycobacteria in macrophages. Significant positive correlations were observed between actively replicating bacteria and respective persister numbers at **(A)** day 3 and **(B)** day 5. **(C)** No significant difference between bacterial numbers was observed between day 3 and day 5 (Wilcoxon test). **(D)** The median percentage of persisters in relation to the respective intracellular bacterial numbers was assessed. No significant differences in the median percentage of persisters between days 3 and 5 was observed (p = 0.784; Wilcoxon test). Correlations were assessed using the Pearson’s product-moment (linear), or Kendall’s Tau correlation (non-linear). The blue line represents the regression line for the correlation analyses (Pearson and Kendall), while the black line and associated shaded area represents the local non-linear regression and 95% confidence interval. Box and whisker plots express distribution of data, indicating the median (bold line), interquartile range (box), and range (whiskers). The data was independently conducted in 7 biological experiments, including technical triplicates. Significant p-values (p < 0.05) are shown in bold. r^2^, Pearson’s correlation coefficient squared; CI, 95% confidence interval; τ, Tau correlation coefficient.

In support of the results above, a strong positive correlation between actively replicating bacteria and persister numbers was observed at day 3 (p = 2.20e^-16^; **Figure 4A**) and day 5 (p = 4.44e^-16^; **Figure 4B**) following infection. Whilst a strong linear correlation between actively replicating and persister numbers was observed at day 3, the correlation began to skew towards a non-linear relationship at day 5 (**Figure 4C**). Although the increase in actively replicating bacterial numbers was minimal and not significant (p = 0.432; **Figure 4C**), this does indicate continued growth of the actively replicating bacteria throughout the infection (**Figure 2C**, **3**, **4**). Persisters contrastingly possessed similar bacterial numbers between days 3 and 5 (p = 0.321; **Figure 4C**). Whilst persister numbers may appear greater for higher initial infection burdens, a similar median percentage of persisters, which is an absolute count of persisters relative to the overall intracellular burden was observed across all infection burdens from day 3 to day 5 (24.25% to 23.25%; p = 0.784; **Figure 4D**). This likely indicates a fixed frequency of bacteria in the starting population that go on to form persisters, irrespective of the infection burden.

### Persister numbers are influenced by macrophage-associated antimicrobial responses

To determine the impact that selected host processes may have on the ability of *M. tuberculosis*Δ*leuD*Δ*panCD* to enter a persister state, we exploited inhibitors of macrophage phagocytosis and phagosome acidification. Firstly, cytochalasin D (CytD) was used to inhibit F-actin polymerization, a process crucial for phagocytosis. Previous analysis showed that CytD effectively decreased bacterial uptake, inhibited macrophage apoptosis and decreased secretion of pro-inflammatory cytokines (Ding et al., 2005; Basu et al., 2007; Rohde et al., 2007). Further, an inhibitor of the V-ATPase proton pump, Bafilomycin A1 (BafA1), was utilized to inhibit phagosome acidification. BafA1 has been shown to prevent phagosome acidification and autophagy by inhibiting V-ATPase (Simeone et al., 2015; Smyth et al., 2021).

**Figure 5:**
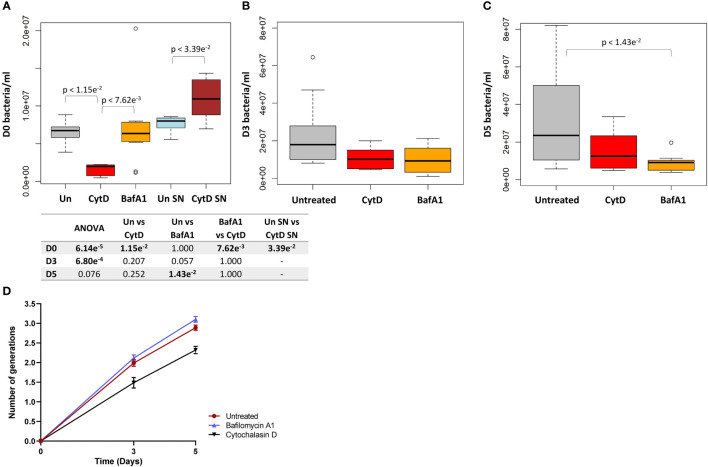
Intracellular bacterial growth following treatment with inhibitors of phagocytosis and phagosome acidification. Macrophages were either untreated or pre-treated with 6 μM CytD or 10 nM BafA1 for 40 min prior to infection with *M. tuberculosis*Δ*leuD*Δ*panCD*::pTiGc pre-induced with 4 mM theophylline. **(A)** Following internalization, macrophages were lysed and intracellular bacteria was harvested for flow cytometry. A 3.42 fold decrease in the median bacterial uptake was detected following CytD treatment compared to untreated macrophages at day 0 (p = 1.15e^-2^). The supernatant following bacterial uptake displayed significantly increased bacterial numbers following CytD treatment (p = 3.39e^-2^), confirming inhibition of phagocytosis of *M. tuberculosis*Δ*leuD*Δ*panCD*::pTiGc. Uptake of bacteria was unaffected by BafA1 treatment compared to bacterial numbers harvested from untreated macrophages (p = 1.000). The effect of the inhibitors on bacterial growth were assessed at **(B)** day 3 and **(C)** day 5 post infection. Bacterial numbers following CytD treatment remained similar to untreated macrophages at day 3 and day 5 (p-values > 0.207), whilst a significant decrease in bacterial numbers following BafA1 treatment was observed at day 5 compared to untreated macrophages (p = 1.43e^-2^). Box and whisker plots express distribution of data independently conducted in biological triplicate, including technical triplicates, indicating the median (bold line), interquartile range (box), and range (whiskers). Significance testing between groups was assessed by repeated measures ANOVA and pairwise Students t-test with Bonferroni correction; significant p-values (p < 0.05) are shown in bold. **(D)** The number of intracellular bacterial generations from day 3 to day 5 for untreated (1.989 ± 0.083 to 2.890 ± 0.066), CytD (1.486 ± 0.133 to 2.320 ± 0.093), and BafA1 (2.115 ± 0.082 to 3.103 ± 0.074) increased over time. Despite lower bacterial numbers recorded following CytD treatment, the number of bacterial generations steadily increased, similarly to the untreated and BafA1-treated group. Plots represent data independently conducted in 4 biological experiments, including technical triplicates, indicating mean ± SEM. Un, untreated; SN, supernatant; SEM standard error of mean.

As expected, following CytD treatment, significantly lower bacterial numbers were recovered from macrophages following internalization compared to untreated samples (p = 1.15e^-2^; **Figure 5A**). The reduced uptake of bacteria was further confirmed by the significantly increased bacterial numbers harvested from the supernatant of CytD-treated macrophages compared to untreated macrophages following bacterial uptake (p = 3.39e^-2^; **Figure 5A**). A 3.42 fold decrease in median bacterial numbers was observed following CytD treatment compared to bacterial numbers in untreated macrophages following internalization, confirming its ability to inhibit phagocytosis (**Figure 5A**).

**Figure 6:**
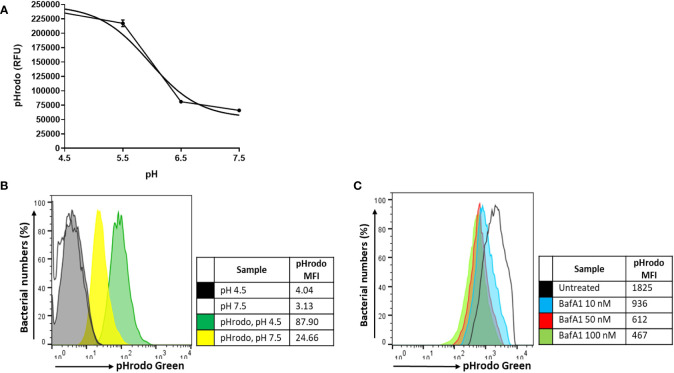
pH calibration of pHrodo Green. **(A)** Exponentially growing *M. tuberculosis*Δ*leuD*Δ*panCD::*pSTCHARGE was labelled with 0.5 mM pHrodo Green, exposed to potassium phosphate buffers ranging between pH 4.5-7.5, and visualized spectrophotometrically. A sigmoidal line of best fit was applied to the graph using GraphPad Prism, generating a pH lookup table to accurately determine the pH (**Supplementary Table 2**). Results are representative of triplicate samples, displaying geometric mean ± SD, and involved the removal of background fluorescence from unstained cells. **(B)** Unfixed macrophages infected with unlabelled or pHrodo-labelled *M. tuberculosis*Δ*leuD*Δ*panCD*::pSTCHARGE pre-induced with 4 mM theophylline were harvested and exposed for 60 min to pH 4.5 and pH 7.5 potassium phosphate buffers to determine whether a change in intracellular fluorescence could be observed using flow cytometry. The increase in pHrodo MFI reflects the decrease in pH. **(C)** Macrophages were pre-treated with increasing concentrations of BafA1 40 min prior to infection with pHrodo-labelled *M. tuberculosis* Δ*leuD*Δ*panCD*::pSTCHARGE pre-induced with 4 mM theophylline. Intact macrophages were harvested following internalization and resuspended in HBSS buffer (unfixed) prior to analysis using flow cytometry. Live cells were gated according to TurboFP635 positivity, thereafter pHrodo fluorescence was assessed. Increasing BafA1 concentrations led to a decrease in pH as detected by the decreasing pHrodo MFI. To minimize possible adverse effects associated with higher DMSO concentrations, 10 nM BafA1 was applied to subsequent experiments. Results are representative of data independently conducted in biological triplicate, including technical triplicates. MFI, median fluorescence intensity; RFU, relative fluorescence units.

A similar initial intracellular bacterial burden was observed between untreated and BafA1-treated macrophages at day 0, confirming that BafA1 treatment did not influence bacterial uptake (p = 1.000; **Figure 5A**). Inhibition of phagosome acidification was confirmed by labelling *M. tuberculosis* Δ*leuD*Δ*panCD* with a lipophilic pH-responsive fluorescent probe, pHrodo Green, which emits increasing fluorescence intensity with a decreasing pH environment (**Figure 6**). pHrodo-labelling was however not suited for monitoring the intracellular pH environment over multiple days due to reduction in pHrodo fluorescence with cell division (result not shown). In addition, formaldehyde fixation greatly reduced pHrodo fluorescence (result not shown). For these reasons, the pH environment could not be assessed throughout infection, although we could confirm reduced phagosome acidification at the start of infection.

The fluorescence dilution reporter was subsequently exploited to calculate the number of bacterial generations at different time points, based on the TurboFP635 MFI. The number of bacterial generations in untreated macrophages increased over 5 days, displaying approximately 2 generations by day 3 (1.989 ± 0.083), and approximately 3 generations by day 5 (2.890 ± 0.066; **Figure 5D**). Despite the intracellular bacterial numbers steadily increasing over 5 days following CytD treatment, the lower number of bacterial generations observed between day 3 (1.486 ± 0.133) and day 5 (2.320 ± 0.093; **Figure 5D**) implies slower bacterial growth in CytD-treated macrophages compared to bacteria from untreated and BafA1-treated macrophages.

**Figure 7:**
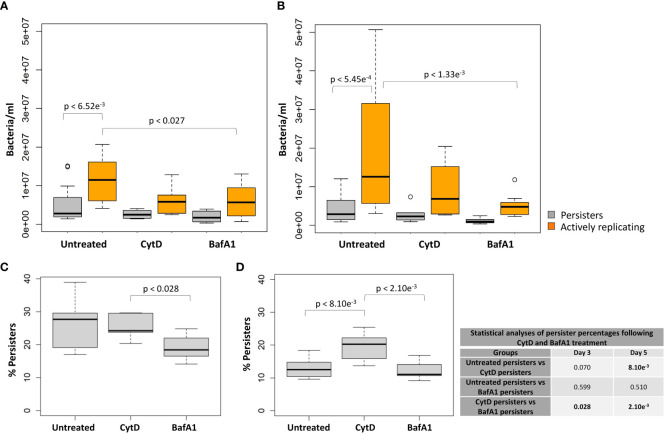
Macrophage antimicrobial processes impact *M. tuberculosis* persister formation. The persister subpopulation was visually assessed using flow cytometry at day 3 and day 5. Significant differences between groups was observed at **(A)** day 3 (p = 0.006) and **(B)** day 5 (p = 0.034; repeated measures ANOVA). p-values are listed in **Table 3**. The median percentage of persisters in relation to the respective intracellular bacterial numbers was assessed at **(C)** day 3 and **(D)** day 5. Significance testing between persister percentages was assessed using a pairwise Students t-test (unpaired) with Bonferroni correction; significant p-values (p < 0.05) are shown in bold. Box and whisker plots express distribution of data independently conducted in 4 biological experiments, including technical triplicates, indicating the median (bold line), interquartile range (box), and range (whiskers).

Significantly higher median numbers of actively replicating bacteria compared to persister numbers were observed in untreated samples at day 3 and day 5 (p-values < 6.52e^-3^; **Table 3**). Contrastingly, no significant differences in median numbers between actively replicating and persister bacteria were observed at and between day 3 and day 5 following CytD treatment (p-values = 1.000; **Figure 7A, B**; **Tables 3**, **4**).

**Table 3:** Comparison of bacterial numbers at day 3 and day 5.

Group 1	Group 2	Day 3 p-value	Day 5 p-value
ANOVA		**0.006**	**0.034**
BafA1 persisters	BafA1 replicating	0.182	1.000
BafA1 persisters	CytD persisters	1.000	1.000
CytD persisters	CytD replicating	1.000	1.000
BafA1 persisters	Untreated persisters	0.539	1.000
CytD persisters	Untreated persisters	1.000	1.000
BafA1 replicating	Untreated replicating	**0.027**	**1.33e^-3^**
CytD replicating	Untreated replicating	0.154	0.191
Untreated persisters	Untreated replicating	**6.52e^-3^**	**5.45e^-4^**

p-values below the statistical significance threshold (p < 0.05) are shown in bold; ANOVA repeated measures and *post-hoc* pairwise Students t-test (unpaired) with Bonferroni correction. p-values below the statistical significance threshold (p < 0.05) are shown in bold.

**Table 4:** Comparison of bacterial numbers between days 3 and 5.

Group 1	Group 2	p-value
ANOVA		**6.877e^-08^**
D3 BafA1 persisters	D5 BafA1 persisters	1.000
D3 BafA1 replicating	D5 BafA1 replicating	1.000
D3 CytD persisters	D5 CytD persisters	1.000
D3 CytD replicating	D5 CytD replicating	1.000
D3 untreated persisters	D5 untreated persisters	1.000
D3 untreated replicating	D5 untreated replicating	**0.039**

p-values below the statistical significance threshold (p < 0.05) are shown in bold; ANOVA repeated measures and *post-hoc* pairwise Students t-test (unpaired) with Bonferroni correction. p-values below the statistical significance threshold (p < 0.05) are shown in bold.

Comparisons of the median bacterial numbers between untreated and CytD-treated macrophages revealed no significant differences for either actively replicating or persister bacteria at day 3 and day 5 (p-values > 0.154; **Figure 7A, B**; **Table 3**). A significantly higher median percentage of persisters was however observed at day 5 following CytD treatment compared to the untreated group (p = 8.10e^-3^; **Figure 7D**), but not at day 3 (p = 0.070; **Figure 7C**; **Table 3**).

Whilst BafA1 treatment did not influence initial bacterial uptake at day 0, (**Figure 5A**), significantly reduced bacterial numbers were recorded at day 5 (p = 1.43e^-2^; **Figure 5C**), but not at day 3, compared to untreated macrophages (p = 0.057; **Figure 5B**). Following BafA1 treatment, an increased number of bacterial generations from day 3 (2.115 ± 0.082) to day 5 (3.103 ± 0.074) was observed, suggesting faster bacterial growth, similarly to bacteria recovered from untreated macrophages (**Figure 5D**).

Actively replicating bacterial numbers were significantly lower following BafA1 treatment compared to untreated bacteria at day 3 (p = 0.027; **Figure 7A**) and day 5 (p = 1.33e^-3^; **Figure 7B**; **Table 3**). The stronger significant association at day 5 could be a direct result of the bacterial loss from the BafA1-treated macrophages. Despite this, no significant difference in median persister numbers (p-values > 0.539; **Figure 7A, B**; **Table 3**) and median percentage of persisters (p-values > 0.510; **Figure 7C, D**) were observed at day 3 and day 5 between untreated and BafA1-treated macrophages. Whilst actively replicating bacterial numbers significantly increased in the untreated group between days 3 and 5 (p = 0.039; **Table 4**), no significant increase in median numbers of actively replicating bacteria or persisters were observed following BafA1 treatment (p-values = 1.000; **Table 4**).

Significantly different intracellular bacterial numbers were observed between CytD and BafA1 groups at day 0 (p = 7.62e^-3^; **Figure 5A**), but not at day 3 or day 5 (p-values = 1.000; **Figure 5B, C**). Despite similar numbers of intracellular bacteria between CytD- and BafA1-treated groups, a significantly higher median percentage of persisters was observed following CytD treatment at day 3 (p = 0.028; **Figure 7C**) and day 5 (p = 2.10e^-3^; **Figure 7D**).

### *M. tuberculosis* persisters possess metabolic esterase activity

To establish the use of CV-AM as a metabolic marker, *M. tuberculosis*::pTiGc was sampled *in vitro* during varying growth states to determine the influence of growth phase on *M. tuberculosis* metabolic esterase activity (**Supplementary Figure 1**). Here, we applied CV-AM staining for assessment of the esterase activity in differentially replicating bacterial populations harvested from macrophages at day 3 and day 5 following internalization. The wide range in distribution of esterase activity is indicative of heterogeneous metabolic activity, whereby bacteria (actively replicating and persisters) may exist in varying metabolic states.

**Figure 8:**
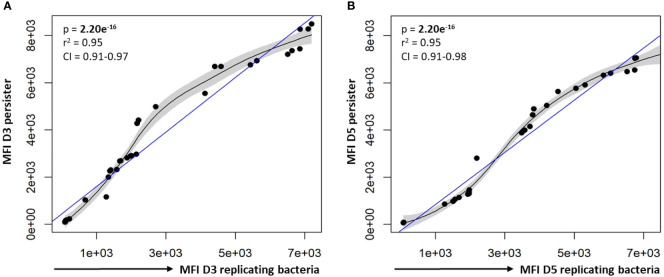
Correlation between the esterase activity of actively replicating bacteria and persisters. The esterase activity of intracellular bacteria was assessed following CV-AM staining and flow cytometric analysis. Significant positive correlations were observed between the esterase activity of actively replicating bacteria and persisters at **(A)** day 3 and **(B)** day 5. Data was assessed using the Pearson’s product-moment correlation (linear) and is representative of data independently conducted in 7 biological experiments, including technical triplicates. Significant p-values (p < 0.05) are shown in bold. The blue line represents the regression line for the correlation analyses (Pearson), while the black line and associated shaded area represents the local non-linear regression and 95% confidence interval. r^2^, Pearson’s correlation coefficient squared; CI, 95% confidence interval; MFI, median fluorescent intensity.

For the different bacterial burdens assessed, a significant positive correlation between the MFI of CV-AM for persisters and actively replicating bacteria was observed at day 3 and day 5 (p-values = 2.20e^-16^; **Figure 8**). As a result, there were no significant differences between the esterase activity of actively replicating bacteria and persisters at day 3 and day 5 (p-values > 0.188; **Supplementary Figure 2**). Furthermore, no significant difference in the esterase activity for actively replicating bacteria (p = 0.136) and persisters (p = 0.854) was detected between days 3 and 5 (**Supplementary Figure 2**).

**Figure 9:**
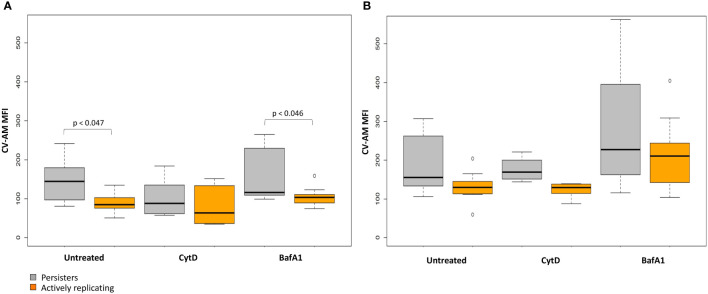
Metabolic esterase activity of persisters is influenced by macrophage antimicrobial processes. The MFI of CV-AM from the actively replicating and persister subpopulations was assessed at day 3 and day 5. No significant differences between groups was observed at **(A)** day 3 (p = 0.198), **(B)** whilst significant differences between groups was observed at day 5 (p = 7.35e^-8^; repeated measures ANOVA). Significance testing between groups is listed in **Table 5** and 6. Box and whisker plots express distribution of data independently conducted in 4 biological experiments, including technical triplicates, indicating the median (bold line), interquartile range (box), and range (whiskers). MFI, median fluorescent intensity.

Repeated measures ANOVA analysis revealed significant differences in the esterase activity of mycobacteria from untreated, CytD- and BafA1-treated groups at day 5 (p = 7.35e^-8^; **Table 5**) and between days 3 and 5 (p = 8.86e^-9^; **Table 6**). Persister bacteria displayed a significant increase in esterase activity compared to actively replicating bacteria for the untreated (p = 0.047) and BafA1-treated group (p = 0.046) at day 3, whilst no significant difference was observed at day 5 (p-values > 0.420; **Table 5**). Furthermore, a significant increase in the esterase activity of persisters was observed from days 3 to 5 following BafA1 treatment (p = 5.22e^-4^; **Figure 9**; **Table 6**).

**Table 5:** Metabolic activity between groups at day 3 and day 5.

Group 1	Group 2	Day 3 p-value	Day 5 p-value
ANOVA		0.198	**7.35e^-8^**
BafA1 persisters	BafA1 replicating	**0.046**	0.420
BafA1 persisters	CytD persisters	0.141	0.159
BafA1 replicating	CytD replicating	1.000	0.461
CytD persisters	CytD replicating	1.000	1.000
BafA1 persisters	Untreated persisters	1.000	0.138
CytD persisters	Untreated persisters	0.557	1.000
CytD replicating	Untreated replicating	1.000	1.000
Untreated persisters	Untreated replicating	**0.047**	1.000

p-values below the statistical significance threshold (p < 0.05) are shown in bold; ANOVA repeated measures and *post-hoc* pairwise Students t-test (unpaired) with Bonferroni correction. p-values below the statistical significance threshold (p < 0.05) are shown in bold.

**Table 6:** Metabolic activity between days 3 and 5.Group 1.

	Group 2	p-value
ANOVA		**8.865e^-09^**
D3 BafA1 persisters	D5 BafA1 persisters	**5.22e^-04^**
D3 BafA1 replicating	D5 BafA1 replicating	**3.02e^-03^**
D3 CytD persisters	D5 CytD persisters	0.502
D3 CytD replicating	D5 CytD replicating	1.000
D3 untreated persisters	D5 untreated persisters	0.696
D3 untreated replicating	D5 untreated replicating	1.000

p-values below the statistical significance threshold (p < 0.05) are shown in bold; ANOVA repeated measures and post-hoc pairwise Students t-test (unpaired) with Bonferroni correction. p-values below the statistical significance threshold (p < 0.05) are shown in bold.

Whilst the esterase activity of persisters in the CytD-treated group was slightly elevated compared to the actively replicating bacteria, this was not significant at day 3 or day 5 (p-values = 1.000; **Figure 9**; **Table 5**). Similarly, the higher esterase activity observed for persisters following BafA1 treatment was not significantly different to persisters following CytD treatment at day 3 and day 5 (p-values > 0.141).

Heterogeneity in esterase activity was observed in persisters and actively replicating bacteria. Together, this shows that persisters possess esterase activity inside macrophages; the change in esterase activity between day 3 and day 5 could further suggest the temporal nature of metabolic processes during intracellular infection.

## Discussion

Diversity in lesion size and bacterial burden have previously been observed in granulomas from non-human primates, where bacilli within the same microenvironment may exist in varying physiological and metabolic states (Lin et al., 2014; Martin et al., 2017). This facilitates the selection of subpopulations more suited to adapt and persist during unfavourable conditions in the host by entering a non- or slowly replicating persistent state, which was effectively observed in macrophage infection models using fluorescence dilution in combination with flow cytometry, as reported in this study and previously (Mouton et al., 2016).

Persister numbers were strongly correlated to the initial infection burden, and the number of intracellular actively replicating bacteria at day 3 and day 5. Irrespective of the varying intracellular bacterial burden in macrophages, this did not lead to significantly different absolute numbers or median percentage of persisters at and between day 3 and day 5.

Since cytokines control macrophage activation and maturation (Marino et al., 2015), it is likely that immune signalling and the antimicrobial activity may be differentially regulated during inhibition of phagocytosis by CytD. Despite significantly lower bacterial numbers observed following inhibition of phagocytosis, CytD treatment resulted in a significantly higher median percentage of persisters at day 5 post infection, compared to bacteria harvested from untreated macrophages. Accordingly, CytD-treated murine macrophages infected with *M. tuberculosis*, and *M. tuberculosis* clinical isolates associated with severe tuberculosis have been suggested to exploit mechanisms to inhibit cytosolic recognition, and subsequently lower host pro-inflammatory cytokine release (Sousa et al., 2020). *M. tuberculosis* furthermore promotes host macrophage differentiation towards the M2 macrophage phenotype, thereby subverting the host immune response by inhibiting inflammatory cytokine release, which enhances intracellular survival and persistence (Refai et al., 2018). *M. tuberculosis* thus appears to use macrophages as residence whilst overcoming host immune evasion strategies.

Following inhibition of phagosome acidification by BafA1 treatment, median persister percentages were not significantly different to untreated macrophages at day 3 and day 5. It is plausible that the reduction in intracellular bacterial numbers observed in our study at day 5 was attributed to extracellular release of *M. tuberculosis* due to continued cell replication and subsequent overburdening of the macrophage. Contrasting reports in the literature indicates that intracellular *M. tuberculosis* numbers are differentially impacted by the infection burden, host cell type and activation state (Lee et al., 2006; Welin et al., 2011; Sullivan et al., 2012; Mehta et al., 2016). Furthermore, *M. tuberculosis* induces an ESX-1-dependent secretion of host pro-inflammatory cytokines as an adaptive strategy to mediate phagosomal rupture and cytosolic escape in mice lungs following treatment with BafA1 (Simeone et al., 2015). Extracellular release of *M. tuberculosis* may have contributed to the significantly reduced actively replicating bacterial numbers harvested from macrophages following BafA1 treatment compared to untreated macrophages at day 3 and day 5.

Genes required for phagosomal exclusion of V-ATPase (2011; Wong et al., 2018), protein synthesis and DNA repair during exposure to acidic pH intracellularly (Vilchèze et al., 2017), may not necessarily be expressed and required during BafA1 treatment. Since the median percentage of persisters were significantly different between CytD- and BafA1-treated groups, this highlights mechanisms whereby *M. tuberculosis* may differentially modulate macrophage metabolic processes and antimicrobial functions (or vice versa), thus affecting the capacity of the host to resolve infection (Gleeson et al., 2016; Martin et al., 2017). The pharmacokinetics of anti-*M. tuberculosis* drugs may furthermore be influenced following BafA1 treatment, as drugs that accumulate in acidic vacuoles possessed a reduced killing capacity (Schump et al., 2017), whilst treatment with isoniazid or rifampicin led to a rapid decrease in drug tolerant *M. tuberculosis* (Mishra et al., 2019; Jain et al., 2020). It would thus be intriguing to determine the influence of these host processes on *M. tuberculosis* gene regulation to provide insight into how *M. tuberculosis* manipulates host immune signals and cytokines to evade immune detection and clearance.

Furthermore, the overall replication rates of intracellular bacteria did not seem to provide an early indication for the onset of persistence, which is in agreement with previous findings where persister formation was shown to be independent of single-cell growth rates (Wakamoto et al., 2013; Raffetseder et al., 2014; Manina et al., 2015). However, variation in *M. tuberculosis* growth rate (Richardson et al., 2016; Nair et al., 2019) and single-cell phenotypic variation (Manina et al., 2015; Rego et al., 2017; Vijay et al., 2017) following exposure to host immune pressures has shown to enhance bacterial phenotypic heterogeneity. Since the pH distribution in macrophages is observed to be heterogeneous (Bhaskar et al., 2014; Dragotakes et al., 2020), it could be valuable to assess how the change in phagosomal pH during infection is linked to the physiological state of *M. tuberculosis* at a single-cell level.

Metabolic adaptation is integral to the intracellular survival and pathogenesis of *M. tuberculosis* (Zimmermann et al., 2017; Fernández-García et al., 2020; Vrieling et al., 2020). CV-AM provided a representation of overall esterase activity in actively replicating bacteria and persisters. Since esterases/lipases are involved in lipid degradation, this provides *M. tuberculosis* with an efficient source of energy and carbon for intracellular persistence (Maurya et al., 2018; Nazarova et al., 2019), as shown using recombinant strains (Lun and Bishai, 2007). Esterases/lipases are furthermore involved in remodelling of cell wall lipids, which have been shown to alter colony morphology, aggregation, and pellicle formation, thereby enhancing *M. tuberculosis* antibiotic tolerance and persistence (Singh et al., 2016; Kumar et al., 2017; Maan et al., 2018).

Overall, an increase in esterase activity was observed from days 3 to 5 for all infection burdens and inhibitor treatments, although this was less pronounced for untreated bacteria. Persisters in the untreated and BafA1-treated group exhibited significantly higher esterase activity compared to actively replicating bacteria at day 3. *M. tuberculosis* from BafA1-treated macrophages additionally demonstrated significantly higher esterase activity for both actively replicating bacteria and persisters from days 3 to 5. It is thus intriguing that although CytD treatment displayed a significantly higher median percentage of persisters at day 3 and day 5, these bacteria appeared to possess lower esterase activity compared to persisters from BafA1-treated macrophages, although this was not significant. This suggests that slowed growth does not account for a reduction in metabolic activity, as previously notioned (Gengenbacher et al., 2010). Literature has shown persisters to exhibit a diversity of metabolic pathways in macrophages (Zimmermann et al., 2017; Huang et al., 2018; Pisu et al., 2020).

The wide distribution in esterase activity observed highlights the dynamic and broad spectrum of metabolic processes that may be activated during differential bacterial replication, in accordance with findings from others (Vijay et al., 2017; Pisu et al., 2020). The change in esterase activity observed for intracellular bacteria from days 3 to 5 could further suggest continued metabolic adaptation to maintain intracellular survival and persistence. This is thought to occur in a temporal nature throughout infection (Rohde et al., 2007; Zulauf et al., 2018), however it has yet to be characterized how adaptation of metabolic pathways is regulated (directly or in response to external signals).

Whilst CV-AM staining may indicate that persisters are metabolically active, detailed characterization of specific *M. tuberculosis* esterases/lipases and their involvement in metabolic functions and adaptation during infection and persistence could prove useful for future work. This could be applied to assess whether specific esterases/lipases differentially influence PDIM production, phospholipid biosynthesis, lipid catabolism and amino acid metabolism during persistence, and how this facilitates bacterial intracellular survival when exposed to lipids of varying chain lengths. Varying chain lengths and trehalose analogues could be assessed for their antigenic potential, as they are suggested to represent novel adjuvants for subunit vaccines (Tima et al., 2017). This could assist in understanding how adaptation promotes remodelling or biosynthesis of specific cell wall components, which could be targeted as an intervention strategy, such as the development of an inhibitor that prevented hydrolysis of the esterase, triacylglycerol (TAG), which inhibited resuscitation of persistent *M. tuberculosis* (Ravindran et al., 2014).

Variation in the composition of *M. tuberculosis* cell wall glycolipids may impact phagocytosis; partial delipidation of the *M. tuberculosis* cell envelope has been shown to enhance host receptor-ligand interactions and phagocytosis (Stokes et al., 2004). Comparatively, over-production of free trehalose led to increased macrophage adhesion, and decreased phagocytosis (Li et al., 2016), whilst overexpression of trehalose dimycolates (TDM, cord factor) promoted macrophage cell death and cytosolic escape (Raffetseder et al., 2019).

## Conclusion

Heterogeneity within *M. tuberculosis* following macrophage uptake, with respect to intracellular bacterial numbers, percentage of persisters, replication dynamics and metabolic esterase activity was observed. The importance of pathogen recognition, phagocytosis, phagosome acidification and maturation as host strategies in inhibiting intracellular growth of *M. tuberculosis* further suggests these host processes as facilitators of persistence. Since adaptation to the host environment is advantageous for intracellular mycobacterial survival, it is unsurprising that the pathogen can exploit multiple routes to achieve this. Coordination between the immunometabolic processes and metabolic remodelling strategies used by *M. tuberculosis* requires further investigation, as heterogeneity at a single-cell level can influence the outcome at a population level. This will not only enhance our understanding of how *M. tuberculosis* interferes with the host immune response, but assist in strategies that effectively target persisters to enhance bacterial clearance.

## Data availability statement

The raw data supporting the conclusions of this article will be made available by the authors, without undue reservation.

## Author contributions

TP, JM and SS conceptualized the experiments. TP performed the experimental work and drafted the manuscript. TP and HS analysed and interpreted results. HS conducted the statistical analysis. HS, JM and SS provided constructive feedback and insightful discussions, and contributed to revising of the manuscript. All authors contributed to the article and approved the submitted version.

## Funding

This work was supported by funding from the South African Medical Research Council (SA MRC), and the South African National Research Foundation (NRF). SLS is funded by the South African Research Chairs Initiative of the Department of Science and Technology and NRF of South Africa, award number UID 86539. HS was funded by Subcommittee C of the research committee at the Faculty of Medicine and Health Sciences of Stellenbosch University. TP was funded by the Deutscher Akademischer Austauschdienst and NRF of South Africa, award number UID 111868. This work was supported by the GCRF Networks in Vaccines Research and Development VALIDATE Network which was co-funded by the MRC and BBSRC (ref MR/R005850/1). This UK funded award is part of the EDCTP2 programme supported by the European Union.

## Conflict of interest

The authors declare that the research was conducted in the absence of any commercial or financial relationships that could be construed as a potential conflict of interest.

## Publisher’s note

All claims expressed in this article are solely those of the authors and do not necessarily represent those of their affiliated organizations, or those of the publisher, the editors and the reviewers. Any product that may be evaluated in this article, or claim that may be made by its manufacturer, is not guaranteed or endorsed by the publisher.

## Supplementary material

Supplementary Figure 1 Varying growth states influence the esterase activity of M. tuberculosis. (A) Metabolic esterase activity of M. tuberculosis::pTiGc was assessed over 21 days in vitro, by staining with CV-AM, followed by (B) flow cytometric analysis. Selecting on live cells according to GFP positivity, the esterase activity was assessed at i) day 2 (early exponential), ii) day 5 (mid exponential), iii) day 13 (late exponential), and iv) day 21 (stationary phase). To distinguish the change in CV-AM MFI, a gate was placed on each normally-distributed population (indicated by the dotted line). Growth curve results are expressed as the mean OD600nm of triplicate samples ± SD. Negligible cell death, as determined by loss of GFP positivity, was observed over 21 days (result not shown). CV-AM: calcein violet AM; MFI: median fluorescent intensity.

Supplementary Figure 2 Heterogeneity in metabolic esterase activity in response to varying bacterial burdens. The distribution of esterase activity was assessed for actively replicating bacteria and persisters at day 3 and day 5. No significant differences between groups was observed at and between day 3 and day 5, as assessed using a pairwise Students t-test (unpaired) with Bonferroni correction. Box and whisker plots express distribution of data conducted in 4 biological experiments, including technical triplicates, indicating the median (bold line), interquartile range (box), and range (whiskers).MFI: median fluorescent intensity.
