# Supplementary figures and images for: Persistence of Mycobacterium tuberculosis in response to infection burden and host-induced stressors
Source: Front Cell Infect Microbiol. 2022 Dec 2;12:981827. doi: 10.3389/fcimb.2022.981827 (PMC9755487; doi:10.3389/fcimb.2022.981827)

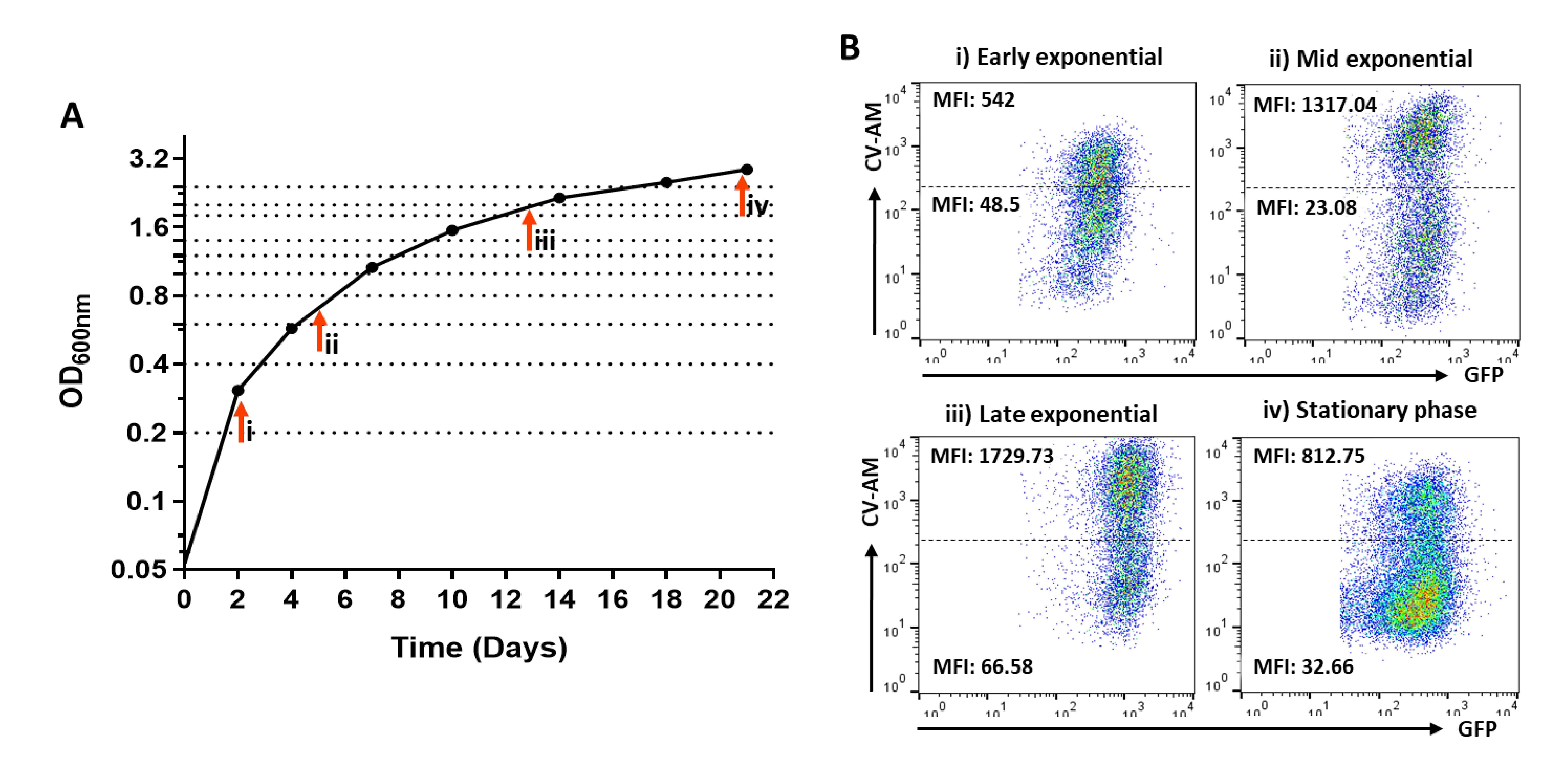

Supplement: Supplementary Figure 1 — Varying growth states influence the esterase activity of M. tuberculosis. (A) Metabolic esterase activity of M. tuberculosis::pTiGc was assessed over 21 days in vitro, by staining with CV-AM, followed by (B) flow cytometric analysis. Selecting on live cells according to GFP positivity, the esterase activity was assessed at i) day 2 (early exponential), ii) day 5 (mid exponential), iii) day 13 (late exponential), and iv) day 21 (stationary phase). To distinguish the change in CV-AM MFI, a gate was placed on each normally-distributed population (indicated by the dotted line). Growth curve results are expressed as the mean OD600nm of triplicate samples ± SD. Negligible cell death, as determined by loss of GFP positivity, was observed over 21 days (result not shown). CV-AM: calcein violet AM; MFI: median fluorescent intensity. [file Image_1.jpeg]

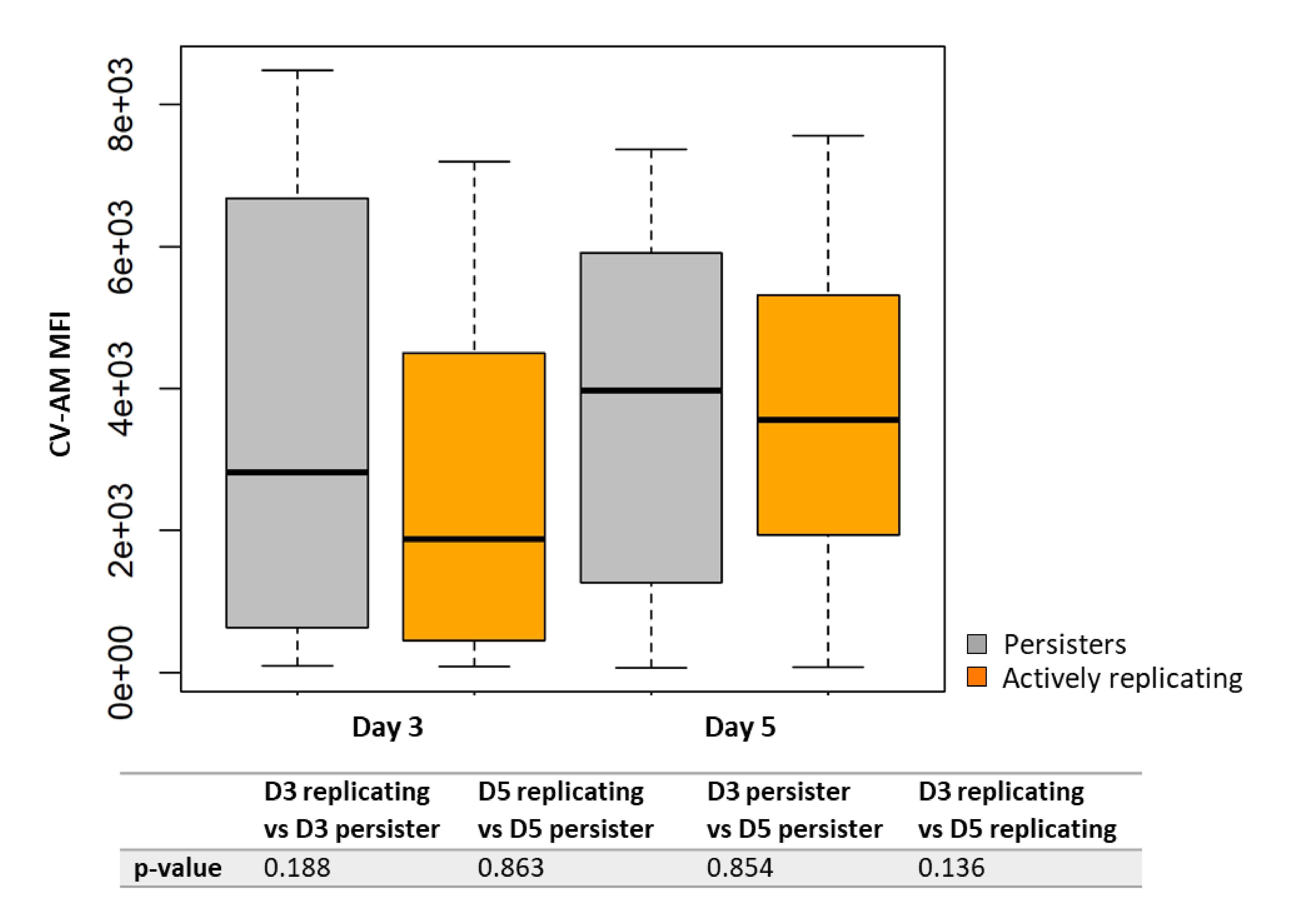

Supplement: Supplementary Figure 2 — Heterogeneity in metabolic esterase activity in response to varying bacterial burdens. The distribution of esterase activity was assessed for actively replicating bacteria and persisters at day 3 and day 5. No significant differences between groups was observed at and between day 3 and day 5, as assessed using a pairwise Students t-test (unpaired) with Bonferroni correction. Box and whisker plots express distribution of data conducted in 4 biological experiments, including technical triplicates, indicating the median (bold line), interquartile range (box), and range (whiskers).MFI: median fluorescent intensity. [file Image_2.jpeg]
