# Supplementary material for: Persistence of Mycobacterium tuberculosis in response to infection burden and host-induced stressors
Source: Front Cell Infect Microbiol. 2022 Dec 2;12:981827. doi: 10.3389/fcimb.2022.981827 (PMC9755487; doi:10.3389/fcimb.2022.981827)
Supplement: Supplementary file 3 [file Table_1.docx]

**Supplementary table 1: Putative esterases/lipases in *M. tuberculosis*.**

| **Esterase** | **Putative functional role** | **Required during stress and/or persistence** | **Subcellular localization** | **References** |
| --- | --- | --- | --- | --- |
| TAG | - Triester of fatty acids combined with glycerol - Carbon and energy source during persistence - Preferentially cleaved by lipases, sometimes by esterases - Reduces toxicity from free fatty acids | Yes | Cell wall and cytoplasm | Sirakova *et al.*, 2012 |
| Culp1 | - Cell wall remodelling, alters lipid composition - Hydrolyses short-medium chain fatty acids - Protective immunity; putative vaccine antigen | Yes | Extracellularly secreted and cell wall | West *et al.*, 2009 |
| Culp4 | - Protective immunity; putative vaccine antigen - Hydrolyses TDM | Yes, nutrient depletion | Cell wall | Yang *et al.*, 2014 |
| Culp3 | - Hydrolyses TDM - Remodels cell envelope to increase nutrient flux | Yes, nutrient depletion | Cell wall | Yang *et al.*, 2014 |
| Culp6 | - Hydrolyses host fatty acids | Yes, hypoxia | Cell wall and membrane | Rastogi *et al.*, 2016; Tallman *et al.*, 2016 |
| Rv1288 | - Mycolyltransferase activity for synthesis of TDM - Cell wall remodelling, alters lipid composition | Yes, nutrient depletion | Cell wall | Maan *et al.*, 2018 |
| PE11, PE16 | - Member of the Lip and PE/PPE family proteins; modulates the host immune response - Hydrolyses short-medium chain fatty acids - Cell wall remodelling, alters lipid composition | Yes, acidic pH, hypoxia and nutrient depletion | Cell wall | Singh *et al.*, 2016; Sultana *et al.*, 2013 |
| Rv3036c | - Preferentially hydrolyses short chain esters - Cell wall remodelling, alters lipid composition | Yes | Cell wall and membrane | Chen *et al.*, 2014; Tallman *et al.*, 2016 |
| Rv0774c | - Hydrolyses short-chain esters - Modulates the host immune response | Yes, iron depletion | Extracellularly secreted | Kumar *et al.*, 2017b |
| Rv0183 | - Putative role in degrading host cell lipids - Involved during reactivation from hypoxia | Yes | Cell wall | Tallman *et al.*, 2016 |
| Rv1683 | - Long-chain acyl CoA synthase - Involved during reactivation from hypoxia | Yes | Cell membrane | Tallman *et al.*, 2016 |
| Rv3591c | - Unknown | Yes, hypoxia | Cell membrane | Ortega *et al.*, 2016; Tallman *et al.*, 2016 |
| Rv0421c | - Unknown | Yes, hypoxia | Cytosol | Ortega *et al.*, 2016; Tallman *et al.*, 2016 |
| Rv0774c | - Cell wall remodelling, alters lipid composition - Modulates the host immune response | Yes | Cell wall | Ortega *et al.*, 2016; Tallman *et al.*, 2016 |
| **Esterase** | **Putative functional role** | **Required during stress and/or persistence** | **Subcellular localization** | **References** |
| Hip1 Rv2224c | - Regulates cleavage and extracellular release of the stress-induced protein GroEL2 - Hydrolyzes medium chain-length ester bonds - Cell wall remodelling, alters lipid composition | Yes, nutrient depletion | Cell envelope | Rengarajan *et al.*, 2008) (Lun and Bishai, 2007 |
| LipC | - Highly immunogenic, stimulates humoral immune and cytokine response - Hydrolyses short-medium chain fatty acids | Yes, nutrient depletion | Cell wall and envelope | Deb *et al.*, 2006; Shen *et al.*, 2012 |
| LipD | - Hydrolyses long chain fatty acids | Yes, oxidative stress and acidic pH | Cell wall and envelope | Gurdyal Singh *et al.*, 2014 |
| LipE | - Hydrolyses medium-chain esters - Putative role in TAG metabolism | Yes, nutrient depletion | Cell membrane | Yang *et al.*, 2019 |
| LipF | - Hydrolyses short chain fatty acids | Yes, acidic pH | Cell wall | Camacho *et al.*, 1999; Zhang *et al.*, 2005 |
| LipH | - Hydrolyses short chain TAG and esters | Yes | Cell wall | Ravindran *et al.*, 2014; Tallman *et al.*, 2016 |
| LipI | - Hydrolyses short chain fatty acids | Yes, nutrient depletion and hypoxia | Cell wall | Lin *et al.*, 2017 |
| LipL | - Hydrolyses short-long chain fatty acids - Induces host immune response | Yes, hypoxia | Cell wall and membrane | Cao *et al.*, 2015; Deb *et al.*, 2006 |
| LipN | - Hydrolyses short chain fatty acids | Yes, acidic pH, hypoxia | Cytosolic | Jadeja *et al.*, 2016 |
| LipU | - Hydrolyses short chain fatty acids | Yes, nutrient depletion | Extracellularly secreted | Kaur *et al.*, 2017; Li *et al.*, 2017 |
| LipV | - Hydrolyses short-medium chain fatty acids | Yes, acidic pH | Cell membrane | Singh *et al.*, 2014 |
| LipY | - Highly immunogenic, regulated by the PE/PPE protein family - Hydrolyses ester bonds in stored TAG (short-long chain fatty acids) - Putative role in resuscitation | Yes, nutrient depletion | Cell wall | Deb *et al.*, 2006; Mishra *et al.*, 2008; Singh *et al.*, 2011 |
| TAG: Triacylglycerol; TDM: Trehalose dimycolate; CULP: Cutinase-like proteins | | | | |
