# Supplementary material for: Persistence of Mycobacterium tuberculosis in response to infection burden and host-induced stressors
Source: Front Cell Infect Microbiol. 2022 Dec 2;12:981827. doi: 10.3389/fcimb.2022.981827 (PMC9755487; doi:10.3389/fcimb.2022.981827)
Supplement: Supplementary file 4 [file Table_2.docx]

| **Supplementary table 2. pHrodo Green pH lookup, 0h** | | | | | | |
| --- | --- | --- | --- | --- | --- | --- |
| **pH** | **pHrodo (RFU)** |  | **pH** | **pHrodo (RFU)** | **pH** | **pHrodo (RFU)** |
| 4,50 | 242107,83 |  | 5,53 | 194951,21 | 6,56 | 91057,28 |
| 4,53 | 241642,84 |  | 5,56 | 192179,86 | 6,59 | 88918,58 |
| 4,56 | 241146,77 |  | 5,59 | 189325,19 | 6,62 | 86871,10 |
| 4,59 | 240617,72 |  | 5,62 | 186390,62 | 6,65 | 84913,77 |
| 4,62 | 240053,72 |  | 5,65 | 183380,20 | 6,68 | 83045,22 |
| 4,65 | 239452,69 |  | 5,68 | 180298,54 | 6,71 | 81263,80 |
| 4,68 | 238812,49 |  | 5,71 | 177150,86 | 6,74 | 79567,58 |
| 4,71 | 238130,85 |  | 5,74 | 173942,89 | 6,77 | 77954,42 |
| 4,74 | 237405,47 |  | 5,77 | 170680,91 | 6,80 | 76422,03 |
| 4,77 | 236633,91 |  | 5,80 | 167371,67 | 6,83 | 74967,92 |
| 4,80 | 235813,71 |  | 5,83 | 164022,36 | 6,86 | 73589,53 |
| 4,83 | 234942,28 |  | 5,86 | 160640,52 | 6,89 | 72284,20 |
| 4,86 | 234017,01 |  | 5,89 | 157234,02 | 6,92 | 71049,19 |
| 4,89 | 233035,22 |  | 5,92 | 153810,95 | 6,95 | 69881,73 |
| 4,92 | 231994,18 |  | 5,95 | 150379,57 | 6,98 | 68779,05 |
| 4,95 | 230891,12 |  | 5,98 | 146948,22 | 7,02 | 67738,37 |
| 4,98 | 229723,27 |  | 6,02 | 143525,23 | 7,05 | 66756,92 |
| 5,02 | 228487,85 |  | 6,05 | 140118,85 | 7,08 | 65831,98 |
| 5,05 | 227182,08 |  | 6,08 | 136737,19 | 7,11 | 64960,87 |
| 5,08 | 225803,25 |  | 6,11 | 133388,09 | 7,14 | 64140,96 |
| 5,11 | 224348,69 |  | 6,14 | 130079,11 | 7,17 | 63369,69 |
| 5,14 | 222815,82 |  | 6,17 | 126817,43 | 7,20 | 62644,57 |
| 5,17 | 221202,18 |  | 6,20 | 123609,81 | 7,23 | 61963,20 |
| 5,20 | 219505,45 |  | 6,23 | 120462,50 | 7,26 | 61323,23 |
| 5,23 | 217723,51 |  | 6,26 | 117381,25 | 7,29 | 60722,43 |
| 5,26 | 215854,44 |  | 6,29 | 114371,27 | 7,32 | 60158,64 |
| 5,29 | 213896,58 |  | 6,32 | 111437,17 | 7,35 | 59629,80 |
| 5,32 | 211848,55 |  | 6,35 | 108582,98 | 7,38 | 59133,92 |
| 5,35 | 209709,31 |  | 6,38 | 105812,14 | 7,41 | 58669,11 |
| 5,38 | 207478,19 |  | 6,41 | 103127,49 | 7,44 | 58233,57 |
| 5,41 | 205154,92 |  | 6,44 | 100531,30 | 7,47 | 57825,58 |
| 5,44 | 202739,67 |  | 6,47 | 98025,27 | 7,50 | 57443,51 |
| 5,47 | 200233,10 |  | 6,50 | 95610,57 |  |  |
| 5,50 | 197636,38 |  | 6,53 | 93287,85 |  |  |
| RFU: relative fluorescence units | | | | | | |
